# Engineering properties as a supplement cementitious material of ground copper reduction slag extracted valuable metal from Cu slag

**DOI:** 10.1016/j.heliyon.2024.e34139

**Published:** 2024-07-04

**Authors:** Jin-Man Kim, Sun-Mi Choi, Sang-Chul Shin

**Affiliations:** a Department of Green Smart Architectural Engineering, Kongju National University, 1223-24, Cheonan-daero, Cheonan, Chungnam, Republic of Korea; b Concrete from Slag with Multifunction Technology, 1223-24, Cheonan-daero, Cheonan, Chungnam, Republic of Korea; c Eco-friendly Concrete Research Center, Kongju National University, 1223-24, Cheonan-daero, Cheonan, Chungnam, Republic of Korea

**Keywords:** Copper reduction slag, Tobermorite, Hydrothermal condition, Supplementary cementitious materials, Grinding efficiency

## Abstract

We have examined whether the copper reduction slag (CRS) generated after recovering valuable metals from copper slag (CS) by reduction process can be used as supplementary cementitious materials (SCMs). According to the test results, the Cu secondary slag with low Fe, Cu, and heavy metal contents had a suitable oxide composition for using as a SCM. CRS showed better grinding efficiency than that of ground blast furnace slag (GGBS). Ground CRS contributed to the formation of tobermorite under autoclaved curing conditions. The compressive strength of CRS mortar replacing 50 % of OPC generated 93 % of that of the OPC mortar. Based on the results of this study, we found that the CRS has highly appropriate engineering characteristics for using as SCMs for concrete. In addition, it is judged that the method of using secondary slag as a material for precast concrete produced under hydrothermal conditions can greatly contribute to the construction process of buildings by securing mechanical performance.

## 1 Introduction

The metal industry has traditionally depended on the mining of natural resources. However, the use of recycled resources has been on the rise due to the rising price of natural resources and stricter environmental regulations on mining. Scrap metal recycling using electric furnaces has therefore become an important process for supplying low and medium-quality steel materials, and the slag generated in the process of refining and steel making of iron is actively used as a substitute for cement or as construction aggregate [1]. Within industries related to steel, the most widely used metal on earth, there are relatively many up-cycling technologies and relevant infrastructure developed for high-value-added recycling of used scrap metal or by-products generated during the manufacturing process [2,3]. However, the up-cycling technology in the non-ferrous metal industry, in which a relatively smaller amount of scrap metal or by-product is generated, remains in the development stage of the original technology [4,5].

Korea relies entirely on imports for copper ore, a raw material for the copper refining process. Waste copper and copper slag generated during the copper refining process can be used as raw materials for metal refining or as SCMs. The amount of copper slag produced in Korea is about 700,000 tons per year [6]. Although relatively many studies have been carried out so far, CS recycling technology remains at a low level [7,8]. Most of the recycling is done by changing the size and shape of the slag to be used as ferrous materials for cement, sandblasting materials for derusting, concrete aggregate, etc [9]. Since about 2.2 tons of CS is generated to make 1 ton of copper [10], the slag generation rate in the copper refining industry is higher than that of other smelting industries. Therefore, the utilization of CS has a significant impact on the industrial competitiveness of the copper refining process [11,12].

CS is a typical ferric by-product containing a high amount of Fe oxide of about 40–50 %, so it is possible to recover pig iron from it [13]. It is known that expensive valuable metals can be obtained when pig iron is recovered from CS by reduction process. However, in addition to a large amount of Fe oxide, copper slag contains a trace amount of copper of about 0.3–3.0 %, which is relatively uniformly dispersed throughout the slag [14]. As for the recycling technology, recovering iron and copper together is preferred since recovering copper alone is not economically or technically feasible due to the meager copper content contained in copper slag [15,16]. When a gravity separation method by melting is used during the general valuable metal recovery process, copper and iron can be recovered together in the form of copper dispersed in iron. However, a trace amount of copper dispersed in iron degrades the quality of structural steels, thus lowering the value of the recycled products. For this reason, technology to recover Fe from copper slag for use in structural steel has not been commercialized yet.

Meanwhile, in the processes such as rolling and forging, though the amount used is relatively small when compared to the structural steel, 0.3–0.8 % of Cu is mixed in steel for casting as well. This is because a variety of mechanical properties such as stiffness, hardness, corrosion resistance, and wear resistance of brittle casting steels can be improved only with a small amount of copper mixed in Ref. [17]. Based on these findings, a research team in Korea reported the results of a study on melting and reducing copper slag to produce an alloy for cast iron [18]. In this study, coke (deoxidizer) and CaO (desulphurizer) were added to the copper slag, the mixture was heated to 1600 °C to reduce Fe and Cu simultaneously, and then it was separated into Fe–Cu alloy and slag to recover valuable metals. Copper slag has only been used as cheap aggregate or landfill material thus far, but this is an up-cycling technology that enables the recovery of valuable metals from the copper slag.

This upcycling technology is performed by a reduction process, and copper reduction slag is generated as a by-product of the process. Because approximately 40 %–50 % of Fe and Cu are present in the copper slag in the form of oxides, the recovered Fe–Cu alloy after the reduction process may be equivalent to about 30 % of the amount of copper slag input. The amount of CRS generated in the process of recovering the alloy increases in proportion to the amount of CaO and carbon added as a reducing agent, reaching approximately 70–85 % of the amount of copper slag used. Although it will depend on the efficiency of the reduction process, since the composition of copper reduced slag (CRS) from which valuable metals are removed is basically similar to that of cement-based materials.

Based on this background, this study attempted to examine the possibility of using as SCMs the CRS generated after recovering valuable metals from copper slag. When the CRS is used as SCMs, the vitrification ratio and fineness of the materials are crucial factors affecting its marketability. In this study, the CRS was discharged into the water at room temperature in a molten state, cooled rapidly to be solid into granule, and then finely ground to evaluate its properties as a binder. The chemical composition of the slag was compared before and after recovering valuable metals to clearly identify its properties as a binder; by evaluating the vitrification ratio, grinding efficiency, and heavy metal leaching of the CRS. The potential of the secondary slag as SCMs and expected problems were reviewed.

In this study, as the next step of reviewing for material properties, mechanical review of cement composites using secondary slag was conducted to expand its applicability in the field of building construction. The flow and compressive strength development of the mortar using secondary slag to replace cement in part were compared with cases with mortar using blast furnace slag (GGBS); hydrate analyses were also performed to identify the characteristics for SCMs. In particular, each test specimen in the mortar tests was cured under atmospheric temperature and autoclave conditions; by comparing the characteristics of individual test specimens, the potential of the CRS for use in the precast concrete products was also examined.

## 2 Experiment plan and materials

### 2.1 Experimental plan

Table 1 shows the experimental plan to evaluate whether the Cu reducing slag (CRS) generated after recovering Fe–Cu alloy from copper slag (CS) has suitable binder performance to be used in the construction industry.

**Table 1:** Experimental plan.

	Test factors	Test levels	Test items
Material properties	Sample types	CS^b^, CRS^c^, GGBS^d^	․ Vitrification ratio ․ Grinding efficiency ․ Heavy metal leaching test
Mixture properties	Binder types	CRS, GGBS	․ Mortar flow ․ Compressive strength ․ Activity index ․ Hydrates analysis (SEM, XRD)
	Substitution ratio to OPC^a^	0, 5, 10, 50 %	
	Curing condition	Air dry curing, Autoclaved curing	

a OPC: Ordinary portland cement.

b CS: Copper slag powder.

c CRS: Copper reducing slag or secondary slag powder extracted Fe & Cu.

d GGBS: Ground granulated blast-furnace slag powder.

The experiment first evaluated the physicochemical properties of the CRS. The items to be evaluated were the vitrification rate, which affects the reactivity, the grinding efficiency, which affects the economic feasibility within the commercialization process, and the heavy metal content, which is an important factor of the products' environmental stability. The values obtained for CRS were then compared with the values of copper slag (CS) discharged from the copper refining process and ground granulated blast furnace slag (GGBS).

Next, the hydration characteristics of the CRS were evaluated with the mortar test specimens. The hydration characteristics analysis was performed targeting mortar mixtures in which the CRS was mixed with 5, 10, and 50 % of the OPC weight; the testing items were fluidity, compressive strength, hydration activity, and hydrate analysis. Hydration activity was evaluated with specimens cured under room temperature and autoclave conditions, and hydrates were evaluated using XRD and SEM. Each experimental result of CRS was evaluated by comparing it with the results of GGBS.

### 2.2 Materials and sample preparations

The copper slag used in this experiment is the slag generated in the refining process of the continuous copper refining method employed by Company G in Korea. In most copper refining plants, including those in Korea, copper slag is treated by a water granulating process in which the slag is dumped into a water tank, in the same way that GGBS is treated [19]. The copper slag used in this study is also in bead-like particles of 5 mm or less, quenched in the water granulating process.

**Fig. 1:**
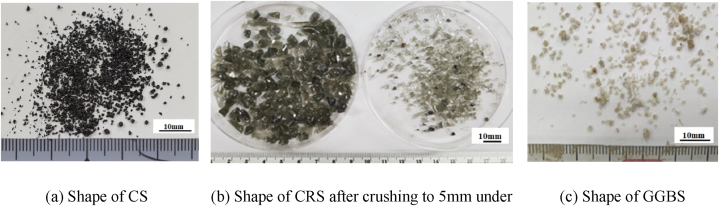
Shape of CS before and after Fe–Cu recovery, GGBS.

**Fig. 2:**
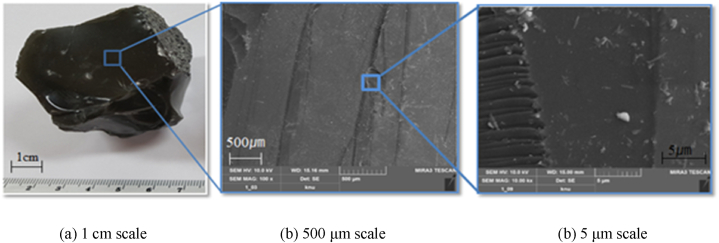
Microstructure on amorphous CRS surface.

Fig. 1 shows the shape of the copper slag, the primary slag (CS) used in the experiment, and the shape of the secondary slag (CRS) generated after recovering Fe from it. The shape of the GGBS is also shown in Fig. 1 for grinding efficiency comparison. As in Fig. 2, showing the secondary slag from which Fe was recovered, the CRS is in an amorphous phase. In this study, this secondary slag was first crushed to a size of 5 mm or less, and then ground using a ball mill. The fine-ground powder was pulverized to have a size similar to that of the blast furnace slag, which is the comparative target for SCMs; the final fineness of the powder was 4347 cm^2^/g.

Ordinary Portland Cement (OPC) with a specific gravity of 3.15 and a fineness of 3,400 cm^2^/g, manufactured by Company H of Korea, was used for mortar mix, and the ground granulated blast furnace slag (GGBS) with 4,227 cm^2^/g fineness was used for the comparation.

For the evaluation of the physical properties of the mortar mixtures with different compositions, mortar specimens were prepared by conducting the mixing process as per KS L ISO 679 standards. Upon completion of the mixing process, the mortar flow was measured, and the specimens for strength test and hydrate analysis were cast. The test items for air-dry curing were air-cured from the day after casting in a constant temperature and humidity room under the temperature of 20 °C and relative humidity of 60 ± 5 % until the date of measurement. Autoclave curing was conducted 3 days after the age of the specimens, heating at a rate of 75 °C/h for 2 h, then maintained at 180 °C and 10 bar for 8 h, followed by cooling at a rate of 30 °C/h for 5 h. The compressive strength test of the specimens after completing the curing process was conducted at room air temperature.

### 2.3 Test methods

The chemical composition of the test items was evaluated using EDX to examine the oxide properties of the slag used in this study. After taking images with SEM, elemental mapping was conducted to look into the dispersion of major constituents of the slag.

The vitrification ratio was quantitatively measured using the Rietveld method with software called SIROQUANT; values were double-checked using a microscopy method [20,21].

For the evaluation of grinding efficiency, the GGBS was used for comparing materials, and pulverizing agents (diethylene glycol series) were added to the ball mill by 0.02 % of the weight of the sample [22]. The powder ground per hour was then collected and the average particle size was measured using Shimadzu's PSA (Laser diffraction particle size analyzer). Using air permeability tests, the fineness of the powder was also measured. To evaluate the potential hazard of the secondary slag when used as construction material, a heavy metal soil leaching test was conducted on the samples before and after the reduction process to recovery valuable metals and measured the Cd, Pb, Zn, As, Cr, and Hg content and pH level of the samples by the relevant Korean standards.

In the physical property evaluation for the mortar mixtures, the mortar flow and compressive strength was measured for each specimen according to KS L ISO 679; the compressive strength of air-dry curing specimens was measured at 3 and 28 days of age, and that of autoclaved curing test specimens was measured after hydrothermal synthesis at 3 days of age after being retained 12 h under 180 °C and 10 bar conditions.

The hydrates were analyzed based on observation and analysis of the cross-section of the samples using Tescan's SEM (Field emission scanning electron microscope); the mineral analysis was conducted in the range of 2θ = 5–60° using Rigaku's XRD (X-ray diffraction).

## 3 Results and discussion

### 3.1 Properties of material

#### 3.1.1 Chemical composition

**Fig. 3:**
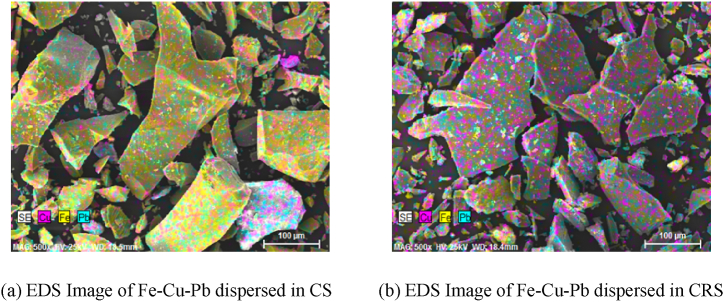
Mapping image of slag before and after Fe–Cu removal.

As can be seen in Table 2 showing the oxide analysis results, CS has a high content of iron oxide of 38.5 %, copper oxide of 2.1 % and manganese oxide of 1.3 %. The element mapping image in Fig. 3 shows that a large amount of Fe and a small amount of Cu were widely dispersed throughout the copper slag generated in the process of copper smelting.

**Table 2:** Physical and chemical properties of binder for hydration test.

Types	Specific gravity	Fineness (blaine, cm^2^/g)	Chemical properties											
			Fe_2_O_3_	SiO_2_	CaO	Al_2_O_3_	ZnO	MgO	CuO	MnO	Na_2_O	SO_3_	Etc.	Sum
OPC	3.15	3400	4.2	17.8	65.8	4.1	0.1	3.0	–	0.2	0.2	2.5	2.1	100
GGBS	2.97	4227	0.5	30.6	44.0	14.0	–	4.4	–	0.3	0.3	4.6	1.3	100
CS	3.50	–	38.5	21.2	16.3	7.8	6.3	2.6	2.1	1.3	1.0	0.2	2.7	100
CRS	2.87	4347	0.7	39.3	32.8	13.6	–	6.3	0.02	1.2	1.7	0.5	3.8	100

However, the total amount of iron oxide, copper oxide, and manganese oxide in CRS from which heavy metals are removed through the reduction process is very small at less than 2 %, and the cast composition is SiO_2_–CaO–Al_2_O_3_, which occupies 85.7 % of the total. The overall composition of CRS is almost similar to that of GGBS.

#### 3.1.2 Vitrification ratio

Most mineral substances form crystals in a stable state at room temperature, but if they are synthesized under special conditions or cooled rapidly before crystallization after melting, constituent particles form an irregular structure similar to a liquid. This is called a non-crystalline (amorphous) or glass phase [23]. It is known that the fine powder in the amorphous phase has better chemical reactivity than the crystalline phase. Therefore, this study attempted to find the vitrification ratio of the fine powder of the slags quantitatively. Two methods were used to measure the vitrification rate of the slag. One is a quantitative method using Rietveld analysis, which quantifies the area of the halo peak based on the XRD analysis result; the other is quantification by measuring the transparency of each particle using an optical microscope image.

The factors that govern the vitrification rate of the slag are the viscosity and cooling rate. The high viscosity lysate has a limited degree of freedom of ionic movement, so it solidifies while maintaining the liquid structure, resulting in a glassy solid state. Conversely, in a low-viscosity lysate, the ion movement has a much higher degree of freedom, so the ions easily crystallize when cooled, resulting in a crystalline solid. The higher the content of a metal oxide such as CaO or FeO, the lower the viscosity and easier crystallization; on the other hand, the higher the SiO_2_ content, the higher the viscosity, making the material suitable to become glassified [24]. As described above, CS contains a large amount of metal oxide, but CRS contains a relatively low amount of metallic oxide and a large amount of SiO_2_. Therefore, it can be estimated that the CRS will show a relatively higher vitrification rate when those two types of slag are quenched in the same manner at the same melting temperature.

**Fig. 4:**
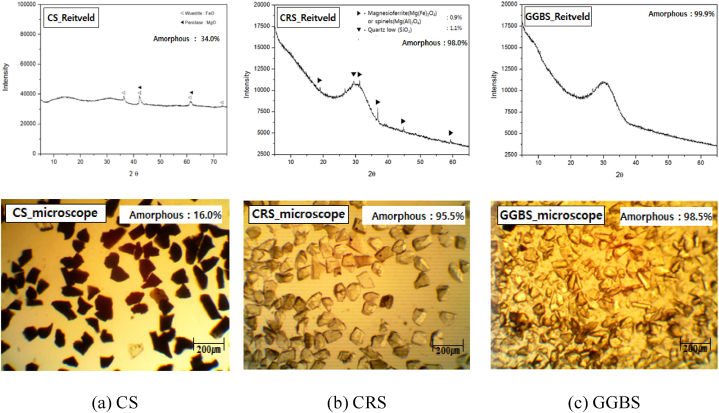
Vitrification ratio and image of CS, CRS, and GGBS by Rietveld and microscope.

Fig. 4 shows the vitrification rate of each type of slag, measured by the Rietveld method and the microscopy method. Since the viscosity values at the starting point of cooling were all similar and the same cooling condition (quenching) was applied, the vitrification rate can be drawn from their oxide compositions. The measured vitrification rates of CRS and GGBS has a high value of 95 % or more, while that of CS was relatively low at 34.0 % even under the same conditions. This is due to the high Fe content, and the Fe content in the molten slag lowers the viscosity of the slag, leading to rapid crystallization at the same cooling rate. These results can be confirmed as similar results in microscopic images. The increase in the vitrification rate is a factor affecting the reactivity activation of cementitious materials. Therefore, when deducing the degree of reaction activation of the secondary slag from its vitrification rate, it seems to be similar to that of GGBS.

The amorphous phase is an unstable compound with no crystal lattices inside, so it becomes transparent thanks to the high light transmittance. Therefore, the vitrification rate can also be measured based on the transmittance of light, observed using a microscope [25]. There are differences in size and number of samples required for evaluation depending on the testing method and country. In this study, 200 particles between 45 and 75 μm in size were selected and their transmittance of light was quantified using an image analysis program. Fig. 4 shows representative images of the analysis. The results of the microscope method confirmed the results from the Rietveld method, showing the CRS vitrification rate at 95.5 %. Based on the above results of amorphous rate evaluation, it was found that CRS can have high reactivity, an important factor to consider when using it as an admixture for cement.

#### 3.1.3 Grinding efficiency

To use the CRS as SCMs, a grinding process is essential. If too much energy is consumed when making the target size fine powder, due to the low grinding efficiency of the material, the economic feasibility of the final products will be low, no matter how excellent the hydration characteristics of the CRS powder. Therefore, materials with not-too-low grinding efficiency are preferred, but it is difficult to determine the ideal level of grinding efficiency quantitatively. Against this backdrop, the grinding efficiency of the CRS was comparatively evaluated with that of quenched GGBS, which is the most widely used SCM now. A batch-type ball mill with a volume of 20 L for laboratory scale was used for the grinding process. Although the grinding efficiency of this equipment is relatively low compared to the continuous type of commercial grinding equipment; this is a widely used method to obtain relative comparison data for each material under the same conditions. The grinding efficiency of the CRS was compared to those of GGBS and CS. Since the GGBS is a commercially available SCM used widely, it can be a yardstick for comparison; the CS was selected because it can help grasp the grinding behavior depending on the presence (or absence) of the metal oxides.

The difference between amorphous and crystalline phases as described above also affects grinding efficiency. The amorphous phase has a relatively unstable structure compared to the crystalline phase, so it is fragile against impact and thus relatively easy to grind. In addition, pulverization is difficult when a material having relatively high toughness (such as Fe oxide) is a component [26,27]. As described above, it is assumed that the CRS will exhibit relatively excellent grinding efficiency due to its higher vitrification rate and lower metal oxide content than those of CS, while presenting grinding efficiency similar to that of GGBS because the two have almost equivalent conditions.

**Fig. 5:**
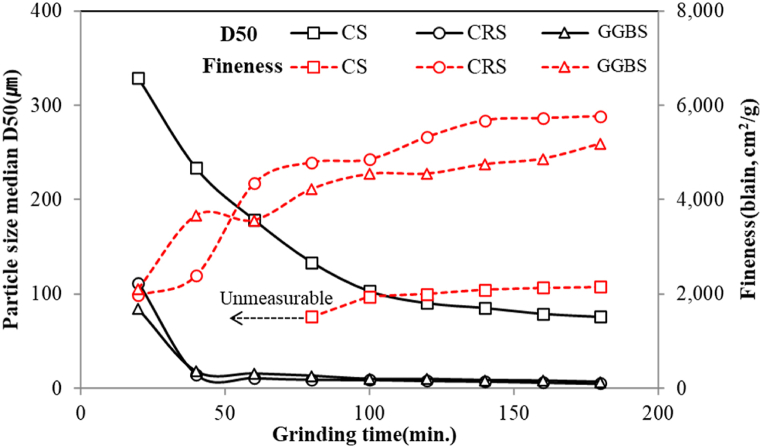
Grinding efficiency of CS, CRS, GGBS.

Fig. 5 shows the experimental results of the grinding efficiency of the CRS, CS, and GGBS. The fineness measured in the ball mill was expressed in the blain value and sizes, having D50 rate with grinding time. As expected, the grinding efficiency of CRS was slightly lower at the beginning than that of GGBS, followed by improved grinding properties afterward. The particle size tended to decrease rapidly from the start to 1 h; it then began to decrease gradually, which is a typical pattern observed in the grinding process. The final results showed that CRS was similar to GGBS in terms of 50 % penetration particle size rate (D50), but the fineness was slightly higher. This is because of the high vitrification rate and low metal oxide content in both types of slag.

As the CRS showed slightly superior grinding properties compared to GGBS, CRS can be used in making products with higher economic feasibility than GGBS. Meanwhile, the grinding efficiency of CS was found to be low due to its lower vitrification rate and a relatively large amount of metal oxides contained compared to the other two types of slag. In particular, the first one and a half hours showed extremely poor grinding efficiency, making it almost impossible to measure the fineness of the material.

#### 3.1.4 Heavy metal leaching test

Since elemental copper is heavier than iron, harmful heavy metals may be left behind during the copper refining process. Among others, the heavy metals may not be sufficiently removed due to relatively low melting temperatures than that of the steelmaking process as the copper refining process is performed at about 1250 °C. On the other hand, the molten metal temperature in the reduction process for Fe–Cu recovery from CS is 1600 °C, making the movement of heavy metals freer and the possibility of heavy metals being contained in the slag relatively low. Meanwhile, the Ministry of Environment of the Republic of Korea (MOE) does not allow wastes containing more than a certain amount of heavy metals to be used for recycling for cement, with rather stricter heavy metal content criteria than that of the leaching method. Table 3 shows the results of heavy metal content measurement carried out in accordance with the MOE standards [28] and standard methods for environmental pollution for the soil in Korea for CS and CRS.

**Table 3:** Results of heavy metal leaching test on CS before and after Fe–Cu recovery.

(Unit: mg/kg)	Cd	Cu	Pb	Cr^6+^	Hg	As	pH
CS	31.4	8100	2510	ND	ND	3.47	8.6
CRS	ND	451	ND	ND	ND	3.78	8.9
Korea Standard on resource for cement	<100	<10,000	<3200	–	<2	<900	–

The results show that although the heavy metal content of CS was within the allowable range of the MOE standards, it was found to contain various types of heavy metal. Hg, Cd, and Cr^6+^ were not detected or stood at safe levels, but Pb and Cu were 2510 mg/kg and 8100 mg/kg, respectively, which were very close to the allowable limits for cement. However, in the case of CRS discharged after collecting Fe and Cu from the CS, the heavy metal content was greatly reduced or not detected at all; Pb was not detected and the Cu content was greatly reduced, presenting highly stable conditions.

**Fig. 6:**
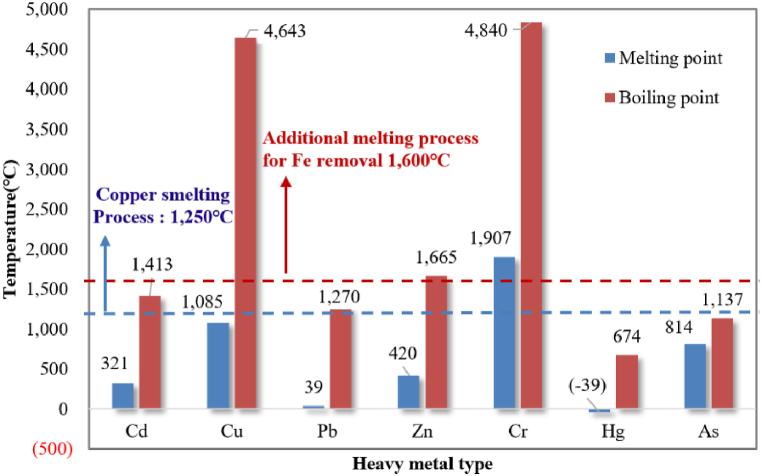
Melting and boiling point according to heavy metal types.

There are various methods for removing a heavy metal, such as the chemical precipitation method, evaporation method, and ion-exchange method; they are selectively utilized in consideration of environmental and economic aspects. In the melting reduction process performed at 1600 °C to recover the Fe–Cu alloy from CS, heavy metal precipitation is actively conducted due to the high temperature of molten metal and, as shown in Fig. 6, proper conditions are created for heavy metals with low vaporizing temperature to be vaporized. This leads to the fairly low content of the six major heavy metals in the CRS, making it suitable for use in construction materials.

In addition, pH level was measured to determine its effect on aging or latent corrosion due to oxidation, which is related to the durability of Copper slag, which needs to be considered when attempting to use it for construction purposes. The pH values of CS and CRS were 8.6 and 8.9, respectively, showing no significant difference.

### 3.2 Properties of slag mixtures

Experiments were conducted to measure the fluidity and hardening properties of CRS using mortar test specimens to determine the material properties when used as SCMs. The comparative material was GGBS, which had similar material properties, and the substitution ratios to cement were set at 5 %, 10 %, and 50 % to examine the characteristics of both cases with small- and large-scale substitution.

#### 3.2.1 Mortar flow

**Fig. 7:**
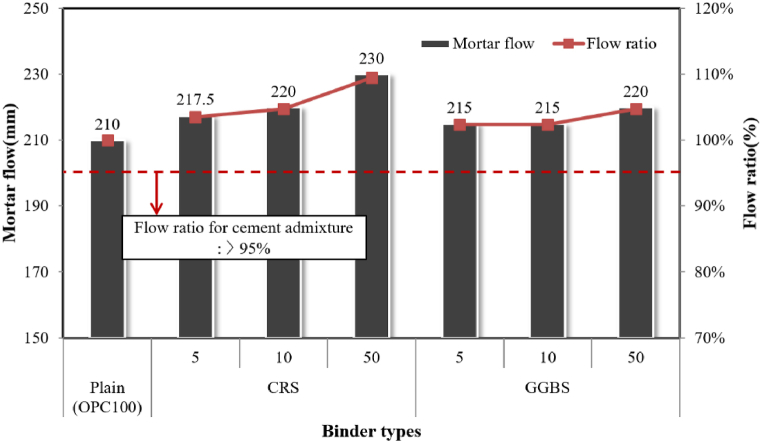
Mortar flow according to binder type.

For the first experiment to evaluate the hydration characteristics of CRS as an admixture for cement, the mortar flow was measured using mortar samples prepared as per the ISO 679 test. In Korea, GGBS, used as an admixture for cement, should meet domestic standards, specified in KS F 2563, Ground granulated blast-furnace slag for use in concrete”. In the standard, a number of criteria that the ground slag should meet to properly perform as admixtures for cement, such as chemical composition, activity index and flow value ratio, etc., are specified. The flow value ratio criteria was 95 % or higher (compared to 100 % OPC samples) for the test samples in which 50 % of the OPC was replaced with GGBS, to ensure workability for GGBS types 1, 2, and 3. Fig. 7 shows the results of the flow test of the mortar in which 5 %, 10 %, and 50 % of OPC was replaced with CRS and GGBS, respectively.

Although both mortar samples using GGBS and CRS showed higher fineness than OPC as the added amount increased, the flow values were higher than that of the plain sample (OPC 100 %) for all substitution ratios, and the fluidity further increased as the ratio increased. Though the flow ratio criterion was 95 %, it increased according to the amount of slag added to the mortar. Notably, the flow reached as high as 220–230 mm when 50 % of cement was replaced with the two types of slag. This characteristic may be driven by the hydrophobicity of the amorphous slag and the way in which the hydration products were formed at the beginning of the hydration reaction. Hydrophobicity decreases the amount of water adhering to the surface of solid particles, thereby increasing the amount of water, which affects fluidity. In addition, the amorphous silica and alumina generated around the slag particles reacting with water rather slowly at the beginning of the hydration reaction form an impermeable coating on the surface of the particles, delaying the hydration reaction, and thus maintaining the fluidity.

#### 3.2.2 Compressive strength

**Fig. 8:**
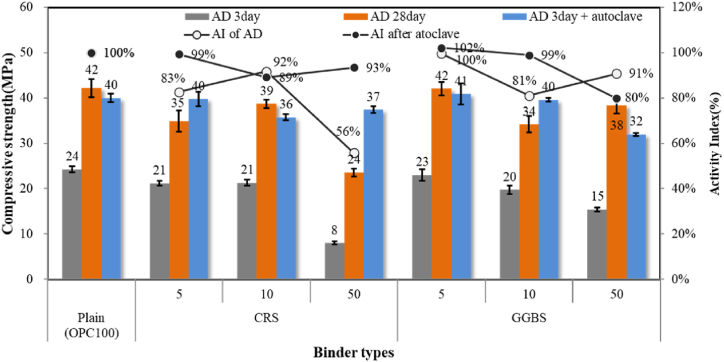
Compressive strength and activity index of mortar.

The mortar specimens produced by replacing 5 %, 10 %, and 50 % of OPC with CRS and GGBS as per ISO 679 were used in the compressive strength tests. The compressive strength of air-dried mortar specimens at 3 and 28 days of age, that of autoclaved mortar specimens at 3 days of age, and the activity index (AI) of each test specimen at 28 days of age were measured, as shown in Fig. 8.

Due to the latent hydraulic properties of amorphous silica contained in GGBS and CRS, when used as SCM in a cement system, the rate of heat generation from hydration and the total heat were known to decrease [29,30]. As a result, the initial strength was also found to be lower than OPC in this experiment.

Regarding the mortar samples with 5 % and 10 % replacement ratios under the air-curing conditions showed that the compressive strength of the CRS mortar was similar to or slightly higher than that of the GGBS mortar. However, for the samples in which OPC was replaced at 50 %, the hydration activity was significantly lower at 56 % for the CRS mortar than the value of 80 % for the GGBS mortar. KS F 2563 (2019) in Korea suggests that the hydration activity of GGBS for concrete should be 75 % or higher on the 28th day and 95 % or higher on the 91st day [31]. Therefore, it may not be appropriate to use CRS in quantities as large as GGBS when the curing is done at room temperature, but only a small amount with a replacement ratio of less than 10 %.

Meanwhile, the CRS has a relatively low CaO content and a high SiO_2_ content compared to GGBS. When manufacturing PC (Pre-cast concrete) products that improve the strength by hydrothermal synthesis through autoclave curing, Si sources are intentionally added to OPC to induce tobermorite generation by hydrothermal synthesis. Tobermorite (C_5_S_6_H_5_) is produced at a relatively high SiO_2_ molar ratio to C–S–H, which is the main hydrate of OPC, so it is used at about 30 %–40 % of the total amount of binder [32]. Since the CRS has a higher SiO_2_ content compared to OPC and a higher CaO content compared to silica powder used as Si sources, it seems to be possible to use it as a source of Si and Ca when manufacturing PC products. To verify this hypothesis, the autoclave-cured mortar test specimens were prepared under the same conditions and their strength was compared with the air-dry cured mortar test specimens on the 3rd day of age, as shown in Fig. 8. As described above, the air-dry curing specimens showed an overall decrease in strength regardless of the CRS replacement ratio, particularly in the 50 % case, presenting a significantly large decrease in strength. The strength decrease rate was greater than that of GGBS in general. However, in the case of autoclave curing, the strength values of mortars with 5 %, 10 %, and 50 % CRS were 99 %, 92 %, and 93 %, respectively, compared to that of plain mortar, indicating that there was no significant decrease in strength. These values are almost the same as those of the case in which the GGBS replacement ratio is low but are much superior to the 80 % hydration rate when GGBS is used in large quantities. Therefore, it is expected that using the CRS for autoclave-cured concrete products will greatly contribute to the saving of the amount of cement used. In the future, our research team will continue our study to determine the optimal C/S mole ratio of CRS to OPC under autoclave curing conditions, as well as CRS application to concrete products.

#### 3.2.3 Hydrates analysis

**Fig. 9:**
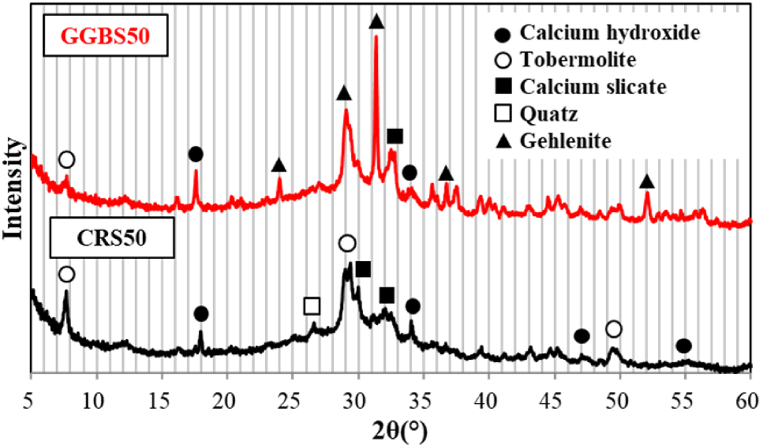
XRD analysis of autoclaved specimen with 50 % CRS and 50 % GGBS.

**Fig. 10:**
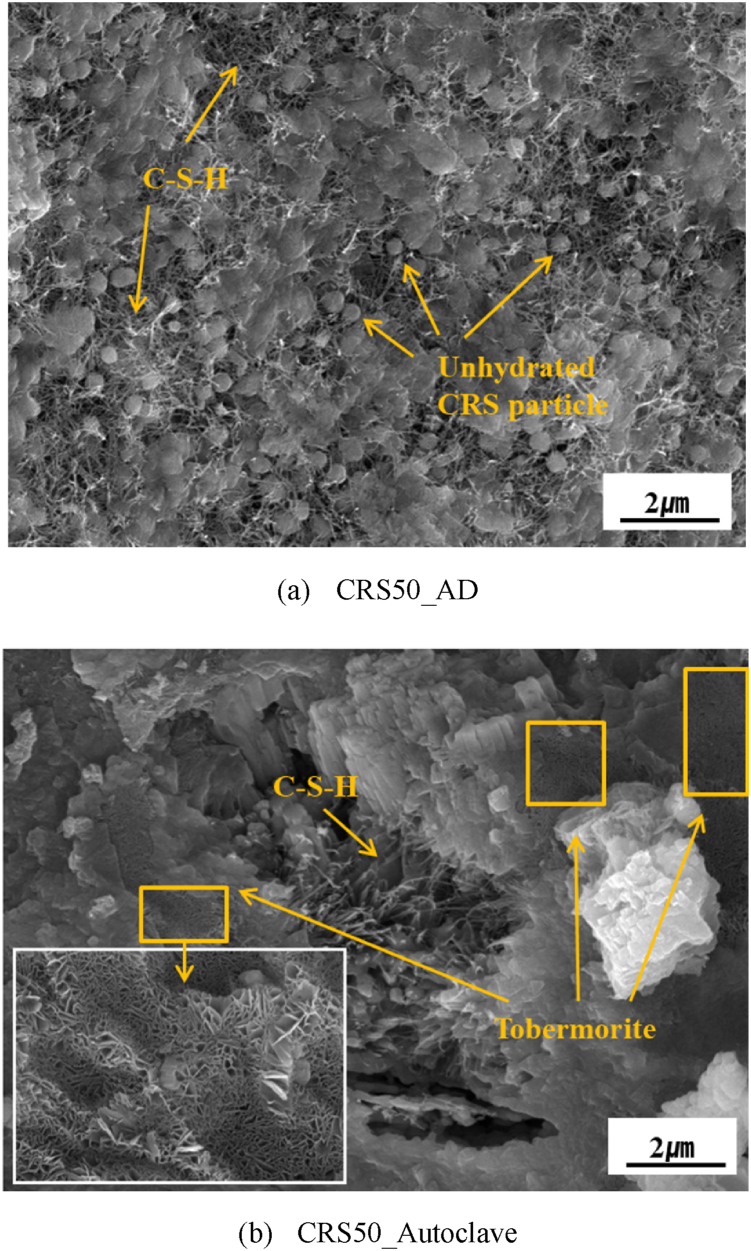
SEM image of CRS50 specimens according curing condition.

The mortar specimens in which 50 % of OPC was replaced with CRS showed a high activity when autoclave cured. To analyze the precise cause of this result, XRD and SEM were measured for hydrates; the results are presented in Fig. 9, Fig. 10.

When the mixture of OPC and GGBS was cured at room temperature, C–S–H, and CH (hydrates of OPC), some AFt or AFm were generated at the beginning of hydration. Subsequently, additional C–S–H is produced by latent hydraulic reaction through alkaline activation of portlandite. Moreover, when a large amount of GGBS is used, the Al_2_O_3_ component leads to the formation of gehlenite hydrate (C2ASH8) [33,34]. When this mixture is autoclaved, it is expected that the reaction between GGBS and tobermorite, a high-temperature hydrate of OPC, is activated, generating gehlenite hydrates.

As predicted, it was found in the experiment that tobermorite and gehlenite hydrates were generated from the hydrates of the autoclave-cured GGBS-OPC mixture. Although the amount of gehlenite hydrate was relatively high, the amount of tobermorite hydrate was less than expected, indicating that the reaction between calcium hydroxide and calcium silicate was not activated. Insufficient supply of Quartz seems to be the cause of this result.

On the other hand, in the hydrates of CRS and OPC mixture, the amount of tobermorite was relatively high, indicating that it contributed to the strength of the hardened specimens, possibly because CRS contains a relatively high content of SiO_2_. However, gehlenite was not produced in CRS-OPC hydrates, and the reaction between calcium hydroxide and calcium silicate was not activated.

These results show that the CRS-OPC mixture exhibits almost the same level of strength as the GGBS-OPC mixture, but shows a different hydration behavior.

The SEM images of the CRS 50 test specimens shown in Fig. 10 indicate that tobermorite marked yellow box is distributed throughout the specimens along with C–S–H hydrates in the autoclave-cured specimens. However, it is easy to see many unhydrated particles between C–S–H hydrates in the air-dry cured specimens. For the CRS-OPC mixture cured at room temperature, therefore, the desired strength may be obtained in the long term of 28 days of age or longer, but it is hard to expect excellent strength within 28 days of age. Based on the results of compressive strength and hydrate analysis, it seems that CRS is more suitable for use as a raw material for precast products in which hardened bodies are produced under hydrothermal synthesis conditions, rather than as a cement admixture for room temperature curing products.

## 4 Conclusions

This study examined the basic chemical properties and the hydration characteristics of secondary copper slag discharged after Fe–Cu recovery from copper slag in the smelting reduction process, to determine its applicability for building construction. The conclusions of this study are as follows:

1) Vitrification rate of CRS showed 98 % or higher values, and the grinding time of it to get the same fineness was shorter than that of GGBS. Additionally, since most heavy metals were not detected, its potential for application as a SCM has been confirmed.
2) The fluidity of the mortar using CRS rose as the amount of slag increased due to its hydrophobicity and impermeable hydrates generated during the initial hydration reaction.
3) The compressive strength of hardened mortar using CRS was found to be similar to that of GGBS mortar when the CRS replacement ratio was small. However, in the high replacement ratio of 50 %, hydration activity of CRS mortar presented as low as 56 %, indicating limitations in the amount of usage for hydration at room temperature.
4) The CRS mortar replacing 50 % of OPC, when cured in autoclave curing, showed 93 % hydration activity, which was much better than the 80 % hydration activity of the OPC-GGBS mortar. Therefore, it was found that the secondary copper slag after recovering valuable metals, such as Fe and Cu from copper slag, is suitable for a raw material in precast concrete products for hydrothermal synthesis.
5) Based on the results of this study, it is judged that the method of using secondary slag as a material for precast concrete produced under hydrothermal conditions can greatly contribute to the construction process of buildings by securing mechanical performance.

## Data availability statement

Data will be made available on request.

## CRediT authorship contribution statement

**Jin-Man Kim:** Writing – review & editing, Validation, Methodology, Conceptualization. **Sun-Mi Choi:** Methodology, Formal analysis, Data curation, Conceptualization. **Sang-Chul Shin:** Writing – original draft, Visualization, Investigation, Data curation.

## Declaration of competing interest

The authors declare that they have no known competing financial interests or personal relationships that could have appeared to influence the work reported in this paper.
